# Persistence of Pneumococcal Serotype 3 in Adult Pneumococcal Disease in Hong Kong

**DOI:** 10.3390/vaccines9070756

**Published:** 2021-07-07

**Authors:** Reema Subramanian, Veranja Liyanapathirana, Nilakshi Barua, Rui Sun, Maggie Haitian Wang, Rita Ng, Edmund A. S. Nelson, David S. Hui, Margaret Ip

**Affiliations:** 1 Department of Microbiology, Faculty of Medicine, The Chinese University of Hong Kong, Prince of Wales Hospital, Hong Kong, China; reema@link.cuhk.edu.hk (R.S.); veranjacl@pdn.ac.lk (V.L.); nilakshibarua@cuhk.edu.hk (N.B.); ritang@cuhk.edu.hk (R.N.); 2 Department of Microbiology, Faculty of Medicine, University of Peradeniya, Peradeniya 20400, Sri Lanka; 3 Centre for Clinical Research and Biostatistics, Jockey Club School of Public Health and Primary Care, The Chinese University of Hong Kong, Hong Kong, China; rsun@gmail.com (R.S.); maggiew@cuhk.edu.hk (M.H.W.); 4 The Seventh Affiliated Hospital of Sun Yat-sen University, Shenzhen 518107, China; 5 Department of Paediatrics, Faculty of Medicine, The Chinese University of Hong Kong, Hong Kong, China; tony-nelson@cuhk.edu.hk; 6 Department of Medicine & Therapeutics, Faculty of Medicine, The Chinese University of Hong Kong, Hong Kong, China; dschui@cuhk.edu.hk

**Keywords:** pneumococcal disease, pneumonia, adults, risk factors, serotype 3

## Abstract

The epidemiology of hospitalised pneumococcal disease in adults following the introduction of universal childhood pneumococcal immunisation in 2009 was assessed. Culture-confirmed *Streptococcus pneumoniae* (SP) from adults hospitalised between 2009 to 2017 were examined. The cases were categorised into invasive pneumococcal disease (IPD) and pneumonia (bacteraemic, non-bacteraemic, and that associated with other lung conditions). The isolates were serotyped and antimicrobial susceptibilities were determined by microbroth dilution. Patient characteristics, comorbidities, and outcomes were analysed. Seven hundred and seventy-four patients (mean age, 67.7 years, SD ± 15.6) were identified, and IPD was diagnosed in 110 (14.2%). The most prevalent serotype, 19F, was replaced by serotype 3 over time. Penicillin and cefotaxime non-susceptibilities were high at 54.1% and 39.5% (meningitis breakpoints), 19.9% and 25.5% (non-meningitis breakpoints), respectively. The overall 30-day mortality rate was 7.8% and 20.4% for IPD. Age ≥ 75 years (OR:4.6, CI:1.3–17.0, *p* < 0.02), presence of any complications (OR:4.1, CI:1.02–16.3, *p* < 0.05), pleural effusion (OR:6.7, CI:1.2–39.4, *p* < 0.03) and intensive care unit (ICU) admission (OR:9.0, CI:1.3–63.4, *p* *<* 0.03) were independent predictors of 30-day mortality. Pneumococcal disease by PCV 13 covered serotypes; in particular, 19F and 3 are still prominent in adults. Strengthening targeted adult vaccination may be necessary in order to reduce disease burden.

## 1. Introduction

Pneumonia accounted for 18.9% of all deaths in Hong Kong in 2019 and has remained the second most common cause of death after cancers since 2012 [1]. Despite the wide availability of pneumococcal vaccines, *Streptococcus pneumoniae* (SP) remains the commonest bacterial pathogen associated with pneumonia and represents 12% of all adult community-acquired pneumonia (CAP) with confirmed aetiologies [2].

The pneumococcal conjugate vaccine, PCV7, was included in Hong Kong’s universal childhood immunisation in 2009 and switched to PCV13 in December 2011. Prior to this time, the vaccine had been available in the private sector. Invasive pneumococcal disease (IPD) was listed as a notifiable infectious disease in 2015 to enhance surveillance. The incidence of IPD has ranged from 1.7–2.9/100,000 population during this postintroduction period [3]. While the incidence among infants has decreased, serotype replacement has been noted, and serotype 3 disease still occurs despite this serotype being included in PCV13.

Since 2014, a single dose of PCV13 followed by a dose of pneumococcal polysaccharide vaccine (PPSV23) one year after PCV13 vaccination has been recommended for high-risk adults and elderly people ≥65 years of age by the Scientific Committee on Vaccine Preventable Diseases under the Centre for Health Protection of Hong Kong. A total of 33.9% and 34.1% of the accumulative percentage of elderly people ≥65 years received at least a single dose of 23-valent pneumococcal polysaccharide vaccine (23vPPV) or PCV13 in 2015–2016 and 2016–2017, respectively. In 2015–2016, elderly people ≥65 years received a single dose of 23vPPV. From October 2017, an additional dose PCV13 has been offered to elderly people ≥65 years who have high-risk conditions [4].

Studies have revealed that childhood immunisation has led to reduced adult pneumococcal disease through herd effects [5]. However, this is less marked in populations with risk factors [6], and disease burden based on IPD surveillance is often underestimated. The efficacy of the adult vaccine for SP is limited in preventing CAP [7]. A recent trial using PCV13 as a vaccine in adults for preventing CAP demonstrated substantial real-world protection of PCV13 against vaccine-type CAP in adults (≥65 years) [8]. Out of 2034 participants, 3.3% were identified as having PCV13 serotypes. However, these participants were less likely to be immunocompromised and overweight or obese than controls but were otherwise similar. The cases were less likely to have received PCV13 than controls.

Patient comorbidities have been independently linked to an increased likelihood of adverse outcomes (e.g., 30-day mortality) [9]. We thus sought to analyse the epidemiology of all culture-confirmed hospitalised pneumococcal disease in adults over a nine-year period and included all disease categories including non-bacteraemic pneumonia or those associated with other lung conditions to study the prevalent serotypes, antimicrobial susceptibilities, and characteristics of the patient groups, associated comorbidities, and outcome.

## 2. Materials and Methods

### 2.1. Patients and Isolates

Adult patients in the age group 16 years to 99 years were reviewed from cases in which SP was isolated during admission to the Prince of Wales Hospital, Hong Kong (PWH) and fulfilled the definition for pneumococcal infection based on the diagnoses from the electronic medical records from January 2009–December 2017. PWH is a 1350-bed teaching hospital and is the major acute hospital for the New Territories East Cluster of the seven public hospital clusters served by the Hospital Authority of Hong Kong. Cases were included based on the confirmed culture of SP and availability of the isolate for serotyping and susceptibility testing. The SP were isolated from sputum (*n* = 670), tracheal aspirate (*n* = 12), blood (*n* = 84), bronchoalveolar lavage (*n* = 5) and others (*n* = 3).

Only the first episode of SP isolation from a patient each year was included. Non-duplicate pneumococcal isolates were serotyped using multiplex polymerase chain reaction (PCR) assays as recommended by the Centre for Disease Control (USA) [10,11]. Microbroth dilution based minimum inhibitory concentration (MIC) determination and interpretation was performed and interpreted according to Clinical and Laboratory Standard Institute (CLSI) guidelines [12]. Intermediate sensitive or resistant isolates were grouped as non-susceptible. Interpretations were also made using European Committee on Antimicrobial Susceptibility Testing (EUCAST) [13] and Pharmacokinetic/pharmacodynamic (PK/PD) guidelines for comparison [14,15].

Clinical data were extracted from medical records, including the electronic patient records (EPR), after discharge. Demographic data including age, sex; administrative data including unit of admission, date of admission, date of discharge; comorbid conditions and disease outcome was collected. Community-acquired infections were defined as developing the symptoms and signs in the community or within 48 h after admission to PWH. Each comorbid condition and its severity were defined based on ICD 9 categories, and the Charlson comorbidity index was calculated [16]. An estimation of the burden of hospitalisation due to infective pneumonia between the period of 2009–2017 at PWH was also retrieved (ICD 9 diagnosis code between 481 to 485). The monthly rates of pneumococcal pneumonia were calculated by dividing against total admission due to infective pneumonia. Pneumococcal disease was broadly defined into six groups (Table 1) to illustrate the clinical characteristics (namely meningitis, bacteraemia, bacteraemic/non-bacteraemic pneumonia, pneumonia with concomitant lung conditions, and exacerbation of lung conditions). The last category was culture positive requiring treatment and with other concomitant lung condition(s), but did not indicate pneumonia in the ICD coding. This group include patients with COPD, emphysema, cancer, etc.

Ethical approval was obtained from the Joint Chinese University of Hong Kong—New Territories East Cluster Clinical Research Ethics Committee (CREC: 2017.448)

### 2.2. Statistical Analysis

Statistical analyses were performed using the forecast package in R [17] and Statistical Package for Social Sciences (SPSS) version 22, IBM, (Chicago, IL, USA). Univariate analysis was conducted by the Fisher’s exact test or Chi-square test for categorical variables and Student’s *t*-test or One-way analysis of variance (ANOVA) for continuous variables.

Multivariate analysis was performed using multiple logistic regression model. *p* < 0.05 was considered statistically significant. The seasonal autoregressive integrated moving average (SARIMA) model was used to forecast the trend of the rate of pneumococcal pneumonia by the R package forecast. The model was prepared using the auto.arima function in the forecast package in R for fitting the best ARIMA model by searching over all possible models within the order constraints. The maximum order of the parameters used was 5, which is the default setting of the auto.arima function. Akaike’s information criterion was used to select the optimal parameters for the SARIMA model. The optimised model is SARIMA(0,1,3)(1,0,0)_12_, where (0,1,3) are the autoregressive order, the degree of differencing, and the moving average order, respectively, and (1,0,0) are the seasonal autoregressive order, the seasonal differencing, and the seasonal moving average order, respectively and the length of the seasonality is represented in subscript (12 months in a year).

## 3. Results

### 3.1. Patient Characteristics

Clinical data of 774 adults hospitalised at PWH during the 9-year period were analysed. Five hundred eighty seven (75.8%) of the patients were male while 24.2% (*n* = 187) were female. The age ranged from 16 to 99 years, with a mean age of 67.7 years (SD ± 15.6). Patients were sorted into four age categories: 12.2% (*n* = 94) between 16–49 years; 23.3% (*n* = 180) between 50–64 years; 28.0% (*n* = 217) between 65–74 years; and 36.6% (*n* = 283) ≥75 years. The demographics and the clinical characteristics are listed in Table 1. The most common comorbidity was hypertension, which was present in 31.0% (*n* = 240) of patients. The proportion of patients with chronic obstructive pulmonary disease (COPD), a history of tuberculosis, hypertension, ischaemic heart disease, congestive cardiac failure and dementia was higher in patients who were ≥75 years. Underlying liver disease was higher in patients between 50–64 years, while the proportion of asthma was higher in patients aged 16–49 years.

### 3.2. Disease and Outcomes

Bacteraemia, meningitis, bacteraemic pneumonia and peritonitis were considered as invasive and accounted for 14.2% (*n* = 110) of the 774 cases. Pneumonia accounted for 61.4% (*n* = 475) of these cases, of which, non-bacteraemic pneumonia accounted for 64.2% (*n* = 305), and pneumonia associated with other lung conditions 17.3% (*n* = 82). The proportion of patients requiring intensive care unit (ICU) admission was 9.09% (*n* = 70 out of 770 cases) (Table 1). The proportion of patients who developed complications was 19.3% (*n* = 149); including respiratory failure (12.4%, *n* = 96,), septic shock (5.94%, *n* = 46), renal failure (3.49%, *n* = 27) and pleural effusions (2.58%, *n* = 20). The 30-day mortality rate was 7.84% (*n* = 60 out of 765 cases). The 30-day mortality was 5.78% (*n* = 38 out of 657 cases) for non-invasive disease and 20.4% (*n* = 22 out of 108 cases) for invasive disease (*p* < 0.001, OR: 4.2, 95% CI: 2.4–7.4).

### 3.3. Serotypes

**Figure 1:**
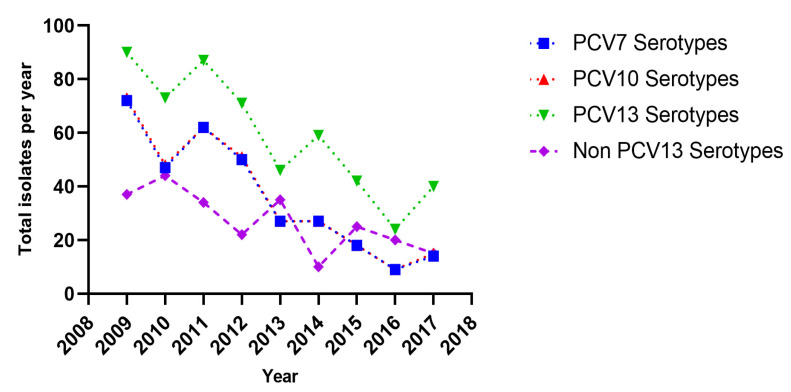
Annual changes in the distribution of the PCV7, PCV10, PCV13 and Non PCV13 serotypes of pneumococci isolated from adult disease.

Out of the 772 isolates serotyped, 19F was the most common type (24.9%, *n* = 192), followed by 3 (17.5%, *n* = 135), serogroup 6 (10.1%, *n* = 78,), 19A (6.22%, *n* = 48), 14 (5.57%, *n* = 43), 23F (5.31%, *n* = 41) and serogroup 15 (4.28%, *n* = 33) (Table 2). Serotype 19F was the commonest serotype amongst the 65–74 and ≥75 age groups. Serotype 3 was the more common amongst the 16–49 and 50–64 age groups. From 2015 onwards 19F was replaced by serotype 3 as the commonest serotype. An increasing rate of serotype 3 was evident (*p* < 0.001) (Table 3). Figure 1 shows the annual changes in the distribution of the PCV serotypes of pneumococci isolated from adult disease.

### 3.4. Antimicrobial Non-Susceptibility

The antimicrobial susceptibilities of 764 SP isolates are listed in Table 4. The penicillin and cefotaxime non-susceptibility rates were 19.9% and 25.5% (non-meningitis breakpoints), 54.1% and 39.5% (meningitis breakpoints), respectively. Non-susceptibilities to erythromycin, tetracycline, chloramphenicol and levofloxacin were 78.9%, 82.5%, 8.25%, and 2.23%, respectively. All isolates were susceptible to vancomycin and linezolid.

The susceptibility rates as interpreted using EUCAST breakpoints and PK/PD criteria are listed in Table 5. For both penicillin and cefotaxime, a much higher percentage of isolates will be resistant due to lower resistance breakpoint. For levofloxacin, 97.8% of the isolates were sensitive (according to PK/PD breakpoints) athough these will be non-susceptible according to EUCAST.

### 3.5. Relationship of Serotypes with Disease/Antimicrobial Non-Susceptibilities

Penicillin, cefotaxime, erythromycin and tetracycline non-susceptibilities had a significant association to serotypes (Table 6). Serotypes 19F and 19A had the highest rates of β-lactam non-susceptibilities of 92.7% and 87.2% to penicillin, 87.0%, and 78.7% to cefotaxime (meningitis breakpoints), respectively. These serotypes were also non-susceptible to erythromycin and tetracycline. All the other serotypes were much more susceptible to β-lactams (non-meningitis breakpoints) and variable susceptibilities to erythromycin and tetracycline. On the other hand, serotype 3 isolates were susceptible to penicillin and cefotaxime at both meningitis and non-meningitis breakpoints.

The relationship between serotypes and invasive disease was statistically significant (Table 6). Invasive disease was primarily accounted for by four serotypes/groups, namely 3, 15, 19A, and 14. The presence of complication was higher in serotypes 19A (37.5%) and 3 (28.9%) disease. Respiratory failure was higher amongst serotypes 3 and 19A disease, and pleural effusion, renal failure and septic shock were higher in serotype 3 disease. After adjusting for potential confounders, including patient age groups and patient comorbidities, neither of the serotypes were independently associated with the presence of complications; however, serotype 3 was independently associated with increased odds of invasive disease (OR:1.7, 95% CI:1.0–2.7, *p* = 0.045).

### 3.6. Thirty-Day Mortality, Patient and Organism Related Factors

The age groups, clinical parameters, comorbidities, common serotypes (Table 2), and antimicrobial non-susceptibilities (susceptible or non-susceptible) were analysed in relation to the 30-day mortality (Table 7). Multivariate analysis revealed that age ≥ 75 years (OR: 4.6, 95% CI:1.3–17.0, *p* < 0.02), presence of any complication (OR:4.1, CI:1.02–16.3, *p <* 0.05), pleural effusion (OR:6.7, CI:1.2–39.4, *p* < 0.03) and ICU admission (OR:9.0, CI:1.3–6.4, *p <* 0.03) were independent predictors of 30-day mortality.

### 3.7. Trends in Hospitalisation Rates Due to Pneumococcal Pneumonia

**Figure 2:**
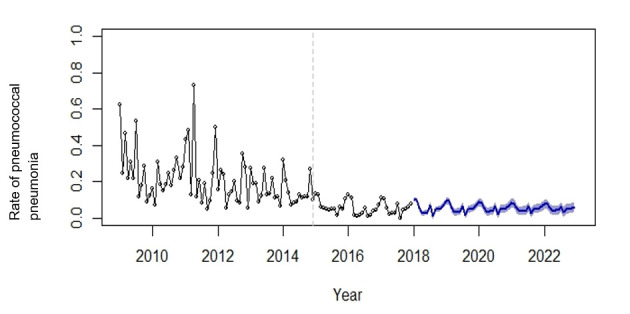
Monthly rate of pneumococcal pneumonia among hospitalised adults at PWH (2009–2017) and projected rates of pneumococcal pneumonia (2018–2022) using SARIMA time series model. Black points represent rates of pneumococcal pneumonia calculated from historical data. Dark blue lines represent projected rates of pneumococcal pneumonia for the following five years. The light blue region represents a 50% confidence interval of the projected rate. The grey dashed line indicates recommendation of PCV13 for high risk adults in Hong Kong (December 2014).

The SARIMA model was prepared to forecast pneumococcal pneumonia hospitalisation, which accounted for 61.4% of the pneumococcal disease in PWH for the span of the 9-year period. Figure 2 shows the time series of pneumococcal pneumonia hospitalisation with respect to all causes infective pneumonia at PWH and trends predicted using the SARIMA model. The mean rate of hospitalisation due to pneumococcal pneumonia declined from 0.209 (SD ± 0.136) prior to adult PCV13 recommendation (on or before December 2014) and 0.060 (SD ± 0.051) after the recommendation (after December 2014) (*p* < 0.001). From the time series data, using the SARIMA model, the predicted rate of pneumococcal pneumonia hospitalisation for the following five years (2018–2022) was also derived. The rate of pneumococcal pneumonia hospitalisation from 2016 to 2017 and the predicted monthly rate for the following five years (2018–2022) show a steady downward trend, with seasonal changes apparent. Two seasonal peaks were present, with a major peak occurring during the winter months (December–February) and a minor summer peak (July), coinciding with the local influenza peaks.

## 4. Discussion

Our data revealed that six of the sixteen comorbid factors, including COPD, a history of tuberculosis, hypertension, ischemic heart disease, congestive cardiac failure, and dementia, were commonly identified in our patients with pneumococcal disease. Individuals with chronic lung and cardiovascular conditions are considered at high risk for pneumococcal disease and are recommended for vaccination. However, hypertension without complications was not considered high risk. In addition, while dementia has not been explicitly listed as a risk factor for pneumococcal disease, there is evidence that people with dementia may be at a greater risk of complications from respiratory infections, including pneumococcal disease [18].

The 30-day mortality due to IPD reported from several other countries ranges from 14% to 20% [19,20,21] which is comparable to that of our IPD (20.4%). The overall 30-day mortality was 7.84%. The ICU admission rate was higher among the two younger age groups. This difference may be attributed to ICU admission policies; however, more complications with septic shock and pleural effusion among the younger patients were identified, thus warranting higher ICU admissions. Differences in the proportions of serotypes in the different age groups may also contribute to the apparent higher proportion of invasive disease and ICU admission among the age groups 16-49 yrs and 50-64 yrs. Serotype 3 was highest amongst the these age groups, which on its own, was independently associated with increased odds of invasive disease.

While serotype 19F was mainly associated with patients ≥ 65 years, from 2015 onwards, serotype 3 has replaced 19F as the most common serotype overall. There have been other reports of serotype 3 remaining the leading cause of invasive disease in adults, despite childhood PCV13 immunisation [22,23]. The persistence of serotypes 19F and the rise of serotype 3 in disease indicate that the herd effect of childhood vaccination may not be as apparent, thus prompting the strengthening of targeted adult vaccination. A Canadian study reported similar findings, where vaccine-covered serotypes still posed a significant burden in adult community-acquired pneumonia as well as invasive disease despite childhood PCV13 vaccination [24]. The increasing rate of serotype 3 related disease locally may be due to clonal expansion, contributing to its survival and persistence despite vaccine pressure and its ability to cause more severe disease.

Furthermore, PCV13 may not be as effective in protecting against serotype 3 disease compared to other serotypes [25]. Serotype 3 may release its capsule, which is not covalently linked to peptidoglycan, thereby evading antibody-mediated protection [26]. In addition, these strains were often susceptible to β-lactam agents. This is consistent with previous findings [27]. While generally comparable, some discrepancies between CLSI and EUCAST breakpoints exist, as exemplified by susceptibilities to a penicillin (non-meningitis) and levofloxacin. Such discrepancies may be problematic, especially for the comparison of data between different regions.

The herd effect of childhood vaccination on adults may be jeopardised due to an increase in disease by non-vaccine serotypes, as exemplified in a recent study [28]. In the current cohort, serogroup 15 was common among non-PCV13 types in adult disease. With previous reports on the increase in carriage of serogroup 15 isolates in children from [29,30] it is evident that serogroup 15 is circulating within the community and is likely to increase in adult disease in the future. Vaccines with expanded serotype coverage, such as the PCV20 (PCV13 types + serotypes 8, 10A, 11A, 12F, 15B, 22F, 33F) has successfully undergone phase 2 trial in adults and has exhibited promising opsonophagocytic activity (OPA) geometric mean fold rises (GMFRs) in functionality antibody from baseline to 1 month after vaccination. PCV20 has shown a GMFR of 37.1, 49.3, 11.2, 113.4, 57.1, 55.4 and 14.0 against serotype 8, 10A, 11A, 12F, 15B, 22F and 33F, respectively. Therefore, PCV20 may be necessary to tackle the anticipated rise in disease by non-PCV13 types [31].

The rate of pneumococcal pneumonia hospitalisation showed a downward trend from 2009–2017, possibly reflecting the combination of the herd effect of routine childhood PCV vaccination implemented in 2009 and the direct impact of adult PCV13 in the latter years.

## 5. Conclusions and Implications

In conclusion, despite routine childhood vaccination, PCV13 type pneumococcal disease, particularly serotype 3 disease, remains a burden in the adult population. Disease caused by other serotypes covered by PCV13 remains prevalent within this adult cohort. High rates of serotype 19F disease remain and are mainly responsible for non-invasive disease among the older age groups. Nonetheless, the high antimicrobial non-susceptibilities within this serotype are a cause for concern as it narrows treatment options for the elderly. High percentages of β-lactam and multidrug resistance in pneumococci have similarly been reported from the Asia Pacific region recently [32]. Publicly funded pneumococcal vaccines have been made available for eligible persons and elderly people ≥65 years since October 2017 to enhance vaccine uptake in Hong Kong. Targeted adult vaccination with expanded serotypes vaccines may be a promising approach in reducing the burden of adult pneumococcal disease.

## Figures and Tables

**Table 1:** Age stratified patient and disease characteristics (*p*-values attained by Fisher’s exact test or Chi-square for categorical variables and on-way ANOVA for continuous variables).

	No. of Patients (N = 774)	16–49 Years (N = 94)	50–64 Years (N = 180)	65–74 Years (N = 217)	≥75 Years (N = 283)	*p* Value
	*n* (%)					
Patient Characteristics						
Sex						
Males	587 (75.8)	53 (56.4)	119 (66.1)	186 (85.7)	229 (80.9)	<0.001
						
Females	187 (24.2)	41 (43.6)	61 (33.9)	31 (14.3)	54 (19.1)	<0.001
Comorbid Conditions						
Diabetes	127 (16.4)	7 (7.45)	23 (12.8)	44 (20.3)	53 (18.7)	0.014
Chronic obstructive pulmonary disease (COPD) (N = 755)	198 (26.2)	5 (5.32)	14 (8.00) (N = 176)	69 (33.2) (N = 208)	110 (39.7) (N = 277)	<0.001
Asthma	53 (6.85)	13 (13.8)	12 (6.67)	15 (6.91)	13 (4.59)	0.024
History of tuberculosis (N = 769)	87 (11.3)	9 (9.60)	9 (5.05) (N = 178)	25 (11.6) (N = 215)	44 (15.6) (N = 282)	0.006
Bronchiectasis	45 (5.81)	1 (1.06)	9 (5.00)	15 (6.91)	20 (7.07)	0.144
Hypertension	240 (31.0)	7 (7.45)	35 (19.4)	79 (36.4)	119 (42.0)	<0.001
Ischaemic heart disease	68 (8.79)	2 (2.13)	9 (5.00)	17 (7.83)	40 (14.1)	<0.001
Congestive cardiac failure	31 (4.01)	2 (2.13)	4 (2.22)	6 (2.76)	19 (6.71)	0.035
Cerebrovascular disease	79 (10.2)	1 (1.06)	12 (6.67)	28 (12.9)	38 (13.4)	0.001
Hemiplegia or paraplegia	20 (2.58)	1 (1.06)	3 (1.67)	6 (2.76)	10 (3.53)	0.474
Dementia	28 (3.62)	0	0	3 (1.38)	25 (8.83)	<0.001
Rheumatological diseases	80 (10.3)	5 (5.32)	17 (9.44)	29 (13.4)	29 (10.2)	0.177
Presence of malignancy	155 (20.0)	7 (7.45)	44 (24.4)	45 (20.7)	59 (20.8)	0.009
Liver disease	59 (7.62)	6 (6.38)	25 (13.9)	15 (6.91)	13 (4.59)	0.003
Peptic ulcer disease	42 (5.43)	2 (2.13)	6 (3.33)	12 (5.53)	22 (7.77)	0.087
Chronic renal disease (N = 773)	57 (7.37)	5 (5.32)	13 (7.26) (N = 179)	12 (5.53)	27 (9.54)	0.307
Charlson Comorbidity Score						
Mean score (SD)	2.03 (± 2.43)	0.947 (± 1.77)	2.72 (± 2.99)	2.07 (± 2.26)	2.21 (± 2.28)	<0.001
No. with Score ≥ 1, *n* (%)	569 (73.5)	40 (42.6)	119 (66.1)	175 (80.6)	235 (83.0)	<0.001
No. with Score ≥ 2, *n* (%)	329 (42.5)	17 (18.1)	73 (40.6)	102 (47.0)	137(48.4)	<0.001
Score ≥ 3, *n* (%)	200 (26.2)	9 (9.97)	48 (26.8)	53 (24.7)	90 (32.6)	<0.001
**Disease**						
Disease Characteristics						
Community acquired Infections (CAI) (N = 771)	676 (87.7)	86 (91.5)	154 (86.5)	187 (86.2)	249 (88.3)	0.564
Invasive infections (IPD) *	110 (14.2)	17 (18.1)	33 (18.3)	20 (9.22)	40 (14.1)	0.044
Broad disease category						
Primary Bacteraemia	14 (1.81)	2 (2.13)	5 (2.78)	5 (2.30)	2 (0.71)	0.356
Meningitis	6 (0.78)	1 (1.06)	2 (1.11)	1 (0.46)	2 (0.71)	0.882
Pneumonia	475 (61.4)	64 (68.1)	115 (63.9)	109 (50.2)	187 (66.1)	0.001
Bacteraemic pneumonia	88 18.52)	14 (21.9)	25 (21.7)	13 (11.95.99)	36 (19.3)	0.029
Non-bacteraemic pneumonia	305 (64.2)	45 (70.3)	82 (71.3)	71 (65.1)	107 (57.2)	0.018
Pneumonia with other lung conditions	82 (17.3)	5 (7.8)	8 (7.0)	25 (22.9)	44 (23.5)	0.001
Exacerbation of other lung conditions	125 (26.3)	5 (7.8)	9 (7.8)	56 (51.4)	55 (29.4)	<0.001
Disease Progression						
Presence of any complication	149 (19.3)	21 (22.3)	39 (21.7)	32 (14.7)	57 (20.1)	0.238
Pleural effusion	20 (2.58)	5 (5.32)	8 (4.44)	3 (1.38)	4 (1.41)	0.045
Septic shock	46 (5.94)	10 (10.6)	20 (11.1)	7 (3.23)	9 (3.18)	<0.001
Respiratory failure	96 (12.4)	15 (16.0)	19 (10.6)	24 (11.1)	38 (13.4)	0.514
Lung collapse	9 (1.16)	0	4 (2.22)	0	5 (1.77)	0.097
Renal failure	27 (3.49)	5 (5.32)	9 (5.00)	6 (2.76)	7 (2.47)	0.339
Lung abscess	5 (0.65)	1 (1.06)	3 (1.67)	1 (0.46)	0	0.162
ICU admission (N = 770)	70 (9.09)	17 (18.1)	28 (15.9) (N = 179)	11 (5.12) (N = 215)	14 (4.96) (N = 282)	<0.001
Mean length of hospitalisation (days)	7.71 (SD 10.1)	7.51 (SD 13.6)	8.94 (SD 11.5)	7.16 (SD 8.26)	7.42 (SD 8.69)	0.320
Outcome						
Death at discharge	36 (4.65)	2 (2.13)	10 (5.56)	8 (3.69)	16 (5.65)	0.427
Died within 48 h of admission	10 (1.29)	0	2 (1.11)	2 (0.92)	6 (2.12)	0.387
30-day mortality (N = 765)	60 (7.84)	1 (1.08) (N = 93)	10 (5.59) (N = 179)	15 (6.98) (N = 215)	34 (12.2) (N = 278)	0.002

* 108 had invasive disease (primary bacteraemia, Meningitis, bacteraemic pneumonia) and the remaining two cases had peritonitis as per final diagnosis in the case records.

**Table 2:** Distribution of common serotypes/groups isolated from patients (*p*-values by Fisher’s exact test or Chi-square test).

Common Serotype/Group	Overall N (%)	16–49 Years *n* (%)	50–64 Years *n* (%)	65–74 Years *n* (%)	≥75 Years *n* (%)	*p*-Value
19F	192 (24.9)	13 (13.8)	28 (15.6)	58 (26.7)	93 (32.9)	<0.001
3	135 (17.5)	25 (26.6)	42 (23.3)	33 (15.2)	35 (12.4)	0.001
Serogroup 6 (6A/B/C/D)	78 (10.1)	9 (9.58)	20 (11.1)	24 (11.1)	25 (8.83)	0.813
19A	48 (6.22)	9 (9.58)	11 (6.11)	16 (7.37)	12 (4.24)	0.238
14	43 (5.57)	5 (5.32)	8 (4.44)	9 (4.15)	21 (7.42)	0.372
23F	41 (5.31)	1 (1.06)	11 (6.11)	14 (6.45)	15 (5.30)	0.243
Serogroup (15 A/B/C/F)	33 (4.28)	4 (4.26)	10 (5.56)	9 (4.15)	10 (3.53)	0.774
Others **	202 (26.2)	28 (29.8)	50 (27.8)	52 (23.9)	73 (25.8)	0.793
Vaccine related serogroups						
PCV 7 types	326 (42.2)	25 (26.6)	59 (32.8)	96 (44.2)	146 (51.6)	<0.001
PCV10 types	330 (42.8)	25 (26.6)	59 (32.8)	98 (45.2)	148 (52.3)	<0.001
PCV13 types	532 (68.9)	61 (64.9)	117 (65.0)	153 (70.5)	201 (71.0)	0.420

** Others comprise of serotypes 1, 4, 5, 7F, 9V and 18C.

**Table 3:** Annual changes in the distribution of the major serotypes/groups of pneumococci isolated from adult disease (*p*-values by Fisher’s exact test or Chi-square tests).

	PCV7 Serotypes (N = 326)	PCV10 Serotypes (N = 330)	PCV 13 Serotypes (N = 532)	Non PCV 13 Types (N = 242)	19F (N = 192)	3 (N = 135)	19A (N = 48)	Serogroup 6 (N = 78)	14 (N = 43)	23F (N = 41)	Serogroup 15 (N = 33)
Year (N)	Total Isolates Per Year (%)										
2009 (127)	72 (56.7)	73 (57.5)	90 (70.9)	37 (29.1)	33 (26.0)	14 (11.0)	2 (1.58)	15 (11.8)	12 (9.45)	12 (9.45)	5 (3.94)
2010 (117)	47 (40.2)	48 (41.0)	73 (62.4)	44 (37.6)	28 (23.9)	13 (11.1)	6 (5.13)	18 (15.4)	7 (5.98)	5 (4.27)	4 (3.42)
2011 (121)	62 (51.2)	62 (51.2)	87 (71.9)	34 (28.1)	38 (31.4)	16 (13.2)	8 (6.61)	9 (7.44)	8 (6.61)	7 (5.79)	4 (3.31)
2012 (93)	50 (53.8)	51 (54.8)	71 (76.3)	22 (23.7)	28 (30.1)	19 (20.4)	1 (1.08)	12 (12.9)	4 (4.30)	9 (9.68)	2 (2.15)
2013 (81)	27 (33.3)	27 (33.3)	46 (56.8)	35 (42.3)	16 (19.8)	9 (11.1)	6 (7.41)	9 (11.1)	5 (6.17)	3 (3.70)	8 (9.88)
2014 (69)	27 (39.1)	27 (39.1)	59 (85.5)	10 (14.5)	19 (27.5)	18 (26.1)	10 (14.5)	6 (8.70)	5 (7.25)	3 (4.35)	3 (4.35)
2015 (67)	18 (26.9)	18 (26.9)	42 (62.7)	25 (37.3)	11 (16.4)	17 (25.4)	5 (7.46)	6 (8.96)	1 (1.49)	2 (2.99)	5 (7.46)
2016 (44)	9 (20.5)	9 (20.5)	24 (54.5)	20 (45.5)	8 (18.2)	9 (20.5)	6 (13.6)	0 (0.00)	0 (0.00)	0 (0.00)	1 (2.27)
2017 (55)	14 (25.5)	15 (27.3)	40 (72.7)	15 (27.3)	11 (20.0)	20 (36.4)	4 (7.27)	3 (5.46)	1 (1.82)	0 (0.00)	1 (1.82)
*p* value	<0.001	<0.001	0.001	0.001	0.265	<0.001	0.005	0.135	0.211	0.059	0.220

**Table 4:** Minimum inhibitory concentrations of *Streptococcus pneumoniae* to 10 antibiotics (N = 764).

Antibiotic	MIC Range (µg/mL)	MIC_50_ (µg/mL)	MIC_90_ (µg/mL)	% Non-Susceptible (*n*)	% Sensitive (*n*)	% Intermediate (*n*)	% Resistant (*n*)
Ciprofloxacin	≤0.25–>32	1	2	-	-	-	-
Levofloxacin	≤0.25–>32	1	1	2.23 (17)	97.8 (747)	0.13 (1)	2.09 (16)
Lincomycin	≤0.25–>32	>32	>32	-	-	-	-
Vancomycin	≤0.03–1	0.25	0.5	0	100 (764)	-	-
Cefotaxime (non-meningitis)	≤0.015–8	0.25	8	25.5 (195)	74.5 (569)	8.90 (68)	16.6 (127)
Cefotaxime (meningitis)	≤0.015–8	0.25	8	39.5 (302)	60.5 (462)	14.0 (107)	25.5 (195)
Penicillin (non-meningitis)	≤0.008–8	0.25	4	19.9 (152)	80.1 (612)	19.5 (149)	0.39 (3)
Penicillin (meningitis)	≤0.008–8	0.25	4	54.1 (413)	45.9 (351)		54.1 (413)
Chloramphenicol	≤1–32	2	4	8.25 (63)	91.8 (701)	-	8.25 (63)
Erythromycin	≤0.015–>64	>64	>64	78.9 (603)	21.1 (161)	1.57 (12)	77.4 (591)
Tetracycline	0.03–>32	32	>32	82.5 (630)	17.5 (134)	2.49 (19)	80.0 (611)
Linezolid	≤0.12–2	0.5	1	0	100 (764)	-	-

**Table 5:** Comparison of antimicrobial non-susceptibilities of *Streptococcus pneumoniae* using CLSI, EUCAST and PK/PD breakpoints of 764 isolates.

		CLSI Breakpoints			EUCAST Breakpoints			PK/PD Breakpoints	
Antibiotic	MIC Range (µg/mL)	% Sensitive (*n*)	% Intermediate (*n*)	% Resistant (*n*)	% Sensitive (*n*)	%Intermediate (*n*)	% Resistant (*n*)	% Sensitive (*n*)	% Resistant (*n*)
Ciprofloxacin	≤0.25–>32	-	-	-	-	-	-	-	-
Levofloxacin	≤0.25–>32	97.8 (747)	0.13 (1)	2.09 (16)	0	97.8 (747)	2.23 (17)	97.6 (746)	2.36 (18)
Lincomycin	≤0.25–>32	-	-	-	-	-	-	-	-
Vancomycin	≤0.03–1	100 (764)	0	0	100 (764)	0	0	-	-
Cefotaxime (non-meningitis)	≤0.015–8	74.5 (569)	8.90 (68)	16.6 (127)	60.5 (462)	22.9 (175)	16.6 (127)	-	-
Cefotaxime (meningitis)	≤0.015–8	60.5 (462)	14.0 (107)	25.5 (195)	-	-	-	-	-
Penicillin (non-meningitis)	≤0.008–8	80.1 (612)	19.5 (149)	0.39 (3)	45.9 (351)	34.2 (261)	19.9 (152)	-	-
Penicillin (meningitis)	≤0.008–8	45.9 (351)	-	54.1 (413)	-	-	54.1 (413)	-	-
Chloramphenicol	≤1–32	91.8 (701)	-	8.25 (63)	94.4 (721)	-	5.63 (43)	91.8 (701)	8.25 (63)
Erythromycin	≤0.015–>64	21.1 (161)	1.57 (12)	77.4 (591)	21.1 (161)	1.57 (12)	77.4 (591)	21.1 (161)	78.9 (603)
Tetracycline	0.03–>32	17.5 (134)	2.49 (19)	80.0 (611)	17.5 (134)	2.49 (19)	80.0 (611)	-	-
Linezolid	≤0.12–2	100 (764)	0	0	100 (764)	0	0	-	-

**Table 6:** Association between common serotypes/groups with antimicrobial non-susceptibilities and disease outcomes.

Serotypes/Groups	19F (N = 192)	3 (N = 135) *	6 (N = 78) **	14 (N = 43)	19A (N = 48) ***	23F (N = 41)	15 (N = 33)	
Outcome	*n* (%)							*p* Value
Antibiotic Non-Susceptibility								
Penicillin (non-meningitis)	118 (61.5)	1 (0.76)	1 (1.32)	1 (2.33)	26 (55.3)	0	0	<0.001
Penicillin (meningitis)	178 (92.7)	3 (2.27)	57 (75.0)	38 (88.4)	41 (87.2)	35 (85.4)	15 (45.5)	<0.001
Cefotaxime (non-meningitis)	150 (78.1)	1 (0.76)	4 (5.26)	3 (6.98)	20 (42.6)	9 (22.0)	3 (9.09)	<0.001
Cefotaxime (meningitis)	167 (87.0)	2 (1.52)	25 (32.9)	18 (41.9)	37 (78.7)	31 (75.6)	6 (18.2)	<0.001
Erythromycin	186 (96.9)	72 (54.6)	72 (94.7)	37 (86.1)	44 (93.6)	37 (90.2)	28 (84.9)	<0.001
Tetracycline	183 (95.3)	94 (71.2)	67 (88.2)	22 (51.2)	45 (95.7)	39 (95.1)	25 (75.8)	<0.001
Disease outcomes								
Invasive disease	7 (3.65)	37 (27.4)	2 (2.56)	11 (25.6)	9 (18.8)	3 (7.32)	9 (27.3)	<0.001
Bacteraemia	0	3 (2.22)	0	2 (4.65)	1 (2.08)	1 (2.44)	3 (9.09)	0.026
Meningitis	1 (0.52)	0	0	0	1 (2.08)	0	0	0.210
Bacteraemic pneumonia	6 (3.14)	34 (25.2)	2 (2.56)	9 (20.9)	7 (14.6)	1 (2.44)	6 (18.2)	<0.001
Non-bacteraemic pneumonia	76 (39.6)	61 (45.2)	35 (44.9)	17 (39.5)	19 (39.6)	18 (43.9)	9 (27.3)	0.302
Pneumonia with other lung conditions	9(9.90)	11(8.15)	9(18.8)	9(11.5)	5(11.6)	2(4.88)	2(6.06)	0.423
Exacerbation of other lung conditions	42 (21.9)	4 (2.96)	7 (14.6)	16 (20.5)	3 (6.98)	8 (19.5)	6 (18.2)	<0.001
Presence of complications	29 (15.1)	39 (28.9)	12 (15.4)	7 (16.3)	18 (37.5)	5 (12.2)	7 (21.2)	0.001
Pleural effusions	3 (1.56)	9 (6.67)	0	2 (4.65)	2 (4.17)	0	0	0.049
Respiratory failure	20 (10.4)	29 (21.5)	9 (11.5)	4 (9.30)	10 (20.8)	4 (9.76)	4 (12.1)	0.011
Renal failure	3 (1.56)	13 (9.63)	1 (1.28)	1 (2.33)	2 (4.17)	0	1 (3.03)	0.006
Septic shock	4 (2.09)	19(14.1)	2 (2.56)	2 (4.65)	6 (12.5)	2 (4.88)	2 (6.06)	<0.001
ICU admissions	4 (2.08)	29 (21.5)	6 (7.69)	4 (9.30)	10 (20.8)	2 (4.88)	2 (6.06)	<0.001
Died within 48 h	0	3 (2.22)	0	0	2 (4.17)	0	6 (6.06)	0.061
30 day mortality ****	16 (8.51)	8 (6.02)	3 (3.85)	0	8 (16.7)	2 (4.88)	4 (12.1)	0.096

* Antibiogram for 132 serotype 3 isolates available; ** Antibiogram for 76 serogroup 6 isolates available *** Antibiogram for 47 serotype 19A isolates available **** missing data (4 serotype 19F cases and 2 serotype 3 cases).

**Table 7:** Risk factors associated with 30-day mortality by univariate and multivariate analysis.

Factor	Odds Ratio (95% CI)	Univariate Analysis	Odds Ratio (95% CI)	Multivariate Analysis
		*p* Value		*p* Value
Age ≥ 49 years	8.86 (1.21–6.47)	0.010		
Age ≥ 65 years	2.62 (1.34–5.13)	0.004		
Age ≥ 75 years	2.47 (1.45–4.21)	0.001	4.61 (1.25–17.0)	0.022
Invasive disease	4.17 (2.35–7.38)	<0.001		
Bacteraemic pneumonia	3.64 (1.97–6.73)	<0.001		
Meningitis	12.3 (2.43–62.4)	0.008		
Exacerbation of existing lung conditions	0.166 (0.04–0.691)	0.005		
Presence of complications	5.43 (3.15–9.35)	<0.001	4.08 (1.02–16.3)	0.046
Pleural effusion	4.18 (1.47–11.9)	0.016	6.77 (1.17–39.4)	0.033
Septic shock	3.38 (1.54–7.41)	0.005		
Respiratory failure	3.18 (1.73–5.84)	<0.001		
Lung collapse	6.13 (1.49–25.1)	0.027		
Renal failure	4.77 (1.92–11.9)	0.003		
ICU admission	3.20 (1.63–6.27)	<0.001	9.01 (1.28–63.4)	0.027
Intubation/ventilatory support	2.49 (1.13–6.27)	0.027		
Serotype 19A	2.56 (1.14–5.75)	0.045		
Charlson comorbidity score ≥ 2	3.22 (1.83–5.66)	<0.001		
Charlson comorbidity score ≥ 3	2.35 (1.37–4.03)	0.002		
Presence of malignancy	2.98 (1.72–5.16)	<0.001		
Dementia	3.62 (1.83–5.66)	<0.001		
Congestive cardiac failure	2.37 (0.877–6.43)	0.087		
Hospitalisation within 3 months prior to admission	2.55 (1.49–4.37)	<0.001		

## Data Availability

The data presented in this study are available on request from the corresponding author. The data are not publicly available due to privacy and ethical restrictions.
